# Acceptability of Self-Sampling for Cervical Cancer Screening Among Women Living With HIV and HIV-Negative Women in Limbé, Cameroon

**DOI:** 10.3389/frph.2020.561202

**Published:** 2021-01-14

**Authors:** Amanda J. Pierz, Rogers Ajeh, Norbert Fuhngwa, Judith Nasah, Anastase Dzudie, Relindis Nkeng, Kathryn M. Anastos, Philip E. Castle, Adebola Adedimeji

**Affiliations:** ^1^Department of Epidemiology and Population Health, Albert Einstein College of Medicine, Bronx, NY, United States; ^2^Clinical Research Education, Networking and Consultancy, Yaoundé, Cameroon; ^3^Limbe Regional Hospital, Limbé, Cameroon; ^4^Faculty of Medicine and Biomedical Sciences, University of Yaoundé I, Yaoundé, Cameroon; ^5^Departments of Medicine, Epidemiology and Population Health, and Obstetrics and Gynecology and Women's Health, Albert Einstein College of Medicine, Bronx, NY, United States

**Keywords:** Sub Saharan Africa, cervical cancer, HIV, HPV, self-sampling, LMIC, stigma

## Abstract

**Introduction:** Like many countries in Sub-Saharan Africa, Cameroon has a high burden of cervical cancer and low availability and uptake of screening. Self-collection has the potential to increase the uptake of cervical cancer screening among Cameroon women. This paper explores patient and community insights surrounding self-collection among women living with HIV and HIV[-] women as well as the barriers and facilitators to obtaining and utilizing self-collected specimens in cervical cancer screening programs.

**Materials and methods:** We utilized an exploratory qualitative approach to obtain data through focus group discussions and in-depth interviews during data collection that took place from May to August 2018. A two-stage sampling strategy was used to select 80 women who participated in six focus group discussions and eight in-depth interviews. We utilized the socio-ecological framework to guide data analysis.

**Results:** All participants indicated that self-sampling was an acceptable method of specimen collection and should be offered as an option for cervical cancer screening in Cameroon. Whereas, most women, regardless of HIV status, preferred the option for self-collection, barriers were identified, such as lack of education about self-collection procedure, being uncomfortable, embarrassed or in pain from the procedure, fear of consequences, perceived competence about ability to self-collect and privacy and confidentiality. We also found that HIV-related stigma was a major concern for HIV[-] women that could prevent them from accessing cervical cancer screening integrated within HIV treatment settings.

**Conclusions:** To promote self-collection for cervical cancer screening, educational interventions with both patients and providers are necessary to increase knowledge of and overall willingness to utilize self-collection. Further research is recommended to examine the role of stigma for HIV[-] women in screening locations associated with HIV treatment.

## Introduction

Cervical cancer-related deaths have significantly declined in high income countries (HIC) such as the United States with a 70% decrease from 1955 to 1992 (1). Much of this success can be attributed to extensive development and implementation of cytology screening programs (2). The most common test is a Papanicolaou (Pap) smear in which a physician obtains a specimen from the transformation zone of the cervix to conduct cytological staining and analysis (3). The development of invasive cervical cancer is contingent on persistent infection with human papillomavirus (HPV) in the uterine cervix, therefore a transformation period of 2–7 years allows for ample opportunities to utilize standard clinical interventions to detect, treat, and manage precancerous lesions (4). Given the possibility for nearly complete prevention, the high incidence of and mortality from cervical cancer in low- and middle-income countries (LMIC)—accounting for 88% of global cervical cancer-related mortality—is particularly tragic (5).

Women living with HIV (WLWH) are at increased risk of HPV infection and cervical pre-cancerous squamous intraepithelial lesions (6). Invasive cervical cancer (ICC) is recognized as an AIDS-defining illness with significant implications for women in sub-Saharan Africa (SSA). In Cameroon, cervical cancer incidence is second only to breast cancer among women of reproductive age (7). Cameroon has a population of 6.74 million women ages 15 years and older who are at risk of developing cervical cancer (8). Although the national HIV infection rate in Cameroon has decreased from 12% in 1995 to 4.5% in 2014, there has been an increase of AIDS-defining illnesses and cancers in people living with HIV (PLWH) (9, 10). This can be attributed to the longer survival rates of PLWH as well as a marked change in the epidemiology of cancer in the general population, which has reduced the average cancer incidence age by about 8 years (11). Even with the launching of the National Programme of Vaccination against Human Papillomavirus in Cameroon, the estimated age-standardized incidence rate of cancer of the cervix uteri in Cameroon is 27.7 per 100,000 with 2,356 new cases and 1,546 deaths reported annually (7, 8).

Low individual uptake of cervical cancer screening is a major obstacle to reducing the morbidity and mortality of cervical cancer in Cameroon (12). Data indicates only 19.7% of all women over the age of 18 have ever been screened for cervical cancer (13). Many patient barriers may hinder compliance with screening guidelines, including but not limited to fear of positive results after screening, anxiety and discomfort surrounding the screening procedure, and cost of the test and/or transportation to clinical setting (13–15). As a means to addressing these barriers, the option to self-collect vaginal or cervical samples is being promoted to increase participation in cervical cancer screening in SSA (16).

Prior to introducing the option of self-collecting cervical specimens as a new screening method, there should be sufficient research to determine if the test is acceptable for this target population. To date, there has been limited literature on the acceptability of self-collection among women in Cameroon, as well as comparative research on preferences among women of differing HIV status. In order to address this gap in knowledge, we conducted a qualitative study to assess and compare women's perceptions and preferences for self- vs. provider-collected specimens in the coastal town of Limbé, Cameroon. Our objective was to explore patient and community insights surrounding self-collection among WLWH and HIV[-] women as well as barriers and facilitators to obtaining and utilizing self-collected specimens in cervical cancer screening programs.

## Materials and Methods

### Study Setting and Population

We recruited a purposive sample of WLWH and HIV[-] women attending the Outpatient Department (OPD) at Limbé Regional Hospital in Limbé, Cameroon. Inclusion criteria were aged 25–59 years, confirmed to be living with HIV or HIV[-] at the time of study recruitment, never having undergone cervical cancer screening, no history of invasive cervical cancer, and being willing and able to understand and provide written, informed paper-based consent to participate. Exclusion criteria were not meeting the inclusion criteria, menstrual bleeding at the time of enrollment, pregnancy, abnormalities or non-menstrual bleeding suggestive of invasive cervical cancer, hysterectomy, and/or based on the judgment of the clinicians.

All procedures performed in this study involving human participants were in accordance with the ethical standards of the National Ethics Committee for Human Health Research in Cameroon and the Albert Einstein College of Medicine IRB. As this study is specifically focusing on cervical cancer, only female-identifying participants with a cervix were included. Informed, written consent was obtained from all participants involved in the study.

### Study Design

This study was conducted as part of a larger study assessing HIV prevalence in WLWH and HIV[-] women within the Central Africa International Epidemiology Database to Evaluate AIDS (CA-IeDEA) in Cameroon. In this parent study, participants obtained their study specimen in a private room at Limbe Regional Hospital, in which they were provided a “Just for Me” self-collection kit [Preventive Oncology International, Cleveland, OH, USA]. The kit included written step-by-step instructional guidelines in both French and English on how to self-collect their own sample. The physician then followed this procedure with the collection of a provider-obtained specimen.

The study team utilized an exploratory-descriptive qualitative approach consisting of a two-stage sampling strategy to select participants. In the first stage, we utilized the master list of 877 women represented in the parent study to identify and generate a list of 585 WLWH and 292 HIV[-] women. The selection of focus group discussion (FGD) and in-depth interview (IDI) interviewees was done after the study nurses contacted potential participants, provided information about the study and invited those who were interested to participate in the FGD or IDI.

The second stage was the systematic sampling of 36 WLWH who participated in three FGD sessions and four WLWH who participated in IDI. The process was repeated to select the same number for FGD and IDI among HIV[-] women, with a total of six FGD and eight IDI. Each focus group consisted of between 10 and 12 people grouped together based primarily on HIV status and age categories consisting of 25–35 years, 36–45 years, and ≥46 years. This resulted in six focus groups with 3 of the focus groups consisting of WLWH and three focus groups for HIV[-] women representing each age category. In addition, eight in-depth interviews (four in each category) were held with individual study participants.

### Data Collection

A structured interview guide was developed to enable rapid assessment of individual and group perceptions and experiences regarding knowledge, attitude and practices of cervical cancer screening, and more specifically their experiences with and preferences for self-collected vs. provider-collected specimens. The guide was organized with several open-ended questions around four key themes: (1) knowledge, attitudes and behaviors regarding cervical cancer and associations with HPV infection, (2) personal and structural facilitators and barriers to screening for cervical cancer, (3) perceptions and preferences for self-collection vs. provided collection options for screening, and (4) knowledge, attitudes and behaviors of people in this community to integration of HIV and non-communicable disease (NCD) screening. The purpose of this guide was to gain valuable insights in the context of cultural and normative factors influencing perceptions, behaviors and the degree to which participants consider access to preventive care as an important component of cervical cancer prevention. Additional information was obtained to assess and compare perceptions and preferences for self- vs. health provider-collected biological specimens to understand women's preferences given contextual factors that facilitate or inhibit access to cervical cancer screening for women.

Focus Group Discussions (FGD) and In-Depth Interviews (IDI) were held between May and August 2018 at an easily accessible and unanimously agreed location within the premises of the Out Patient Department (OPD) of the Limbe Regional Hospital that afforded anonymity to respondents as well as the confidentiality of the information provided by participants. The language used for the interviews and focus group discussions was “pidgin,” a colloquial form of the English language that is widely spoken in the area. A team of three trained research assistants (a moderator, note-taker and observer) facilitated each focus group session, while a team of two (a moderator and note-taker) facilitated the in-depth interviews. Each FGD lasted for 90 min on average while IDI lasted an average of 60 min. Each participant received compensation to cover the cost of transportation and other logistics of participation.

### Data Analysis

Several steps were taken to process the data generated from the FGD and IDI. First, each team member listened to the audio recording and cross-checked the audio with the notes taken during the interview to ensure consistency and data quality. Second, the group that facilitated the discussions and interviews held daily debriefing meetings with the larger research team to give and receive feedback in terms of the study's strength and areas to improve for subsequent interviews. Prior to these briefing sessions, other members of the research team who did not participate in the interview listened to the audio recordings to provide feedback. Finally, the transcription of the audio recordings from “pidgin,” the language of communication during the interviews was translated to English. This was done to ensure that those not familiar with pidgin were able to understand the information obtained from the discussions and interviews. Similar to the process used in transcribing, the translation of transcripts from pidgin to English was done by members of the research team who were not part of the group that facilitated the interview/discussion. The transcripts were independently verified and checked for completeness to ensure all personal identifiers had been deleted.

All discussions were recorded with the participants' consent and translated into English for the purposes of analysis. The analytical process began with data immersion in which team members: (i) listened to the audio recording of each interview, (ii) read the field notes taken by the interview team, (iii) read the original transcripts and translations to ensure consistency with all the data sources and ensure familiarity with the data prior to identifying themes and developing a codebook. These steps facilitated the identification and subsequent validation of a priori themes that were initially developed by the lead author. The research team developed a unified coding scheme, which formed the basis for codifying the data. Thereafter, key themes were identified and matrices were developed to discern patterns and relationships amongst the study themes. Our themes were evaluated by all members of the team as well as within our academic network for expert opinions familiar with the study context for validation.

## Results

### Awareness and Acceptability of Self-Sampling

All the participants interviewed were aware of the option to self-sample as a result of their involvement in this study, and participated in self-collection prior to being interviewed. WLWH advised that those with confidence and knowledge surrounding self-sampling should be given the option to choose between self- and provider collected samples. There was acceptance in both groups that the option to self-collect should be available at clinics for women who were able and willing to perform the procedure. One of the WLWH expressed this when she said:

“If some women say they can [collect their specimen] well, then leave them [the option] to do it themselves.” (WLWH, FGD, aged 25–35)

Most WLWH reported that they preferred that providers collect their samples for them, frequently requesting for a nurse to perform the collection. However, the majority of HIV[-] women reported that they preferred self-collection because many felt they were competent enough to be able to collect their own sample. Descriptors used by the women, such as “better,” “easy,” and “simple” to describe self-collection procedure were often indicators of their acceptance of self-sampling, as opposed to “difficult,” “hard to get right,” “embarrassing,” and “confusing,” often used by women who preferred provider-collected sampling.

### Pain and Fear Surrounding the Provider Sampling Procedure

Both WLWH and HIV[-] women indicated some level of fear surrounding the collection procedure. HIV[-] women indicated their fear of pain from the procedure if sampling is done by the provider. One woman in the group of HIV[-] women aged 46–55 shared her insight that self-sampling will not be as painful when she said: “*Because I will be able to do [the procedure] myself, I will not be able to feel the difference.”* While some of the women had previous clinical experiences that were painful, the majority of concerns about pain from HIV[-] were not based on personal experience but rather anxiety about screening, lack of familiarity with the provider, or anecdotal stories from others who had bad experiences with the procedure.

The WLWH group did not have the same concerns regarding potential pain due to the involvement of the provider. Rather, WLWH reported that their fear was based on concern over the testing procedure itself rather than the provider's involvement. In fact, many of their responses revealed that they found their provider to provide a level of comfort during the collection procedure. Some women expressed this feeling when they reported that:

“I prefer to make them collect because I am scared to collect it myself” (WLWH, FGD, aged 36–45)

“We have fear of this procedure so we need assurance from [our providers]” (WLWH, FGD, aged 25–35)

We can also note that among WLWH who indicated they had a supportive provider, they also reported increased confidence in their ability to self-collect. One woman described the importance of having a supportive provider when she indicated that:

“When I went to collect my own [sample] I used the straw. The doctor that was there, and she nodded her head that I did it well.” (HIV[-], FGD, aged 36–45).

### Perceived Competence About Ability to Self-Collect their Own Specimen

The majority of women, regardless of HIV status or age, felt that they did not have the necessary background information required to collect their own sample as indicated in statements such as “*… I do not have the education to collect it”* (WLWH, IDI, aged 36–45) and “*many of them don't know if they are educated enough to know [how to collect]”* (HIV [-], FGD, aged 36–45). Due to the demands of this study alongside standard clinical care in Limbe Regional Hospital, it is possible that women did not receive more than a simple instruction on the procedure. The lack of education may have contributed to the participants' feeling as though they were undereducated on this new procedure, which may reflect the reality of this clinical setting. Participants who indicated their lack of knowledge or education on the self-sampling procedure may have been a reason to prefer provider-collected samples over self-collection. As some women reported:

“I prefer the health worker because I don't know how to collect so I'd rather they collect.” (WLWH, IDI, aged 36–45)

“I prefer the doctor to collect it because the doctor will do more better than me.” (HIV[-], FGD, aged 36–45)

“I will prefer the health provider to do it, because I will not know what to do.” (WLWH, FGD, aged 46–59)

Nearly all of the participants indicated a concern in their ability to successfully collect their own specimen. As the used self-collection swabs look similar to unused swabs, it might be difficult for women to assess if they swabbed properly or collected enough cells for a viable sample. These concerns about the quality of the samples seemed to be consistent among participants regardless of HIV status:

“… I am not able to do it perfectly” (HIV[-], FGD, aged 46–55),

“I do not know whether I have done it fine or not” (HIV[-], FGD, aged 45–55),

“If they [women] *collect it themselves they might not do it well”* (WLWH, IDI),

“They are scared that they will not do it properly” (WLWH, FGD, aged 36–45).

The statements from the participants indicated a correlation between not feeling educated enough about the procedure with their concern about their ability to self-collect. One participant highlighted their lack of familiarity with the procedure as a reason that they would not succeed at self-sampling by saying:

“What if you're the woman that [self-collects] and you try to collect the specimen but you can't find the side it's located.” (WLWH, FGD, aged 25–35)

The concern in their ability to collect quality samples was overall linked to the participants wanting to make sure that they are not responsible for inaccurate clinical information. In the statement “*I don't do it right so it may have wrong results”* (HIV[-], FGD, aged 36–45), this participant indicated a concern in receiving inaccurate results based on her inability to collect her own sample properly.

### Environmental Context and Stressors

In our study setting, cervical cancer screening was conducted at Limbé Regional Hospital, which is known for providing testing and treatment for HIV to women. Within our study community, the stigma of HIV can affect patient outcomes ranging from discriminatory behavior in health services to delays in enrollment by PLWH. Among the HIV[-] group, there were concerns about the community perception about being seen accessing health services in this setting. This was identified by one of the participants as stigma in their community about HIV when she said that:

“In the first place, HIV alone carries a particular stigma. That stigma is what is preventing people out there to come and do [cervical cancer screening].” (HIV[-], FGD, aged 25–35)

This stigma of HIV leads many community members to not access cancer screening because of fear of being seen going into Limbé Regional Hospital. One participant described the fear of being attached to this community stigma when she said that:

“So when you go now [for testing in your community], when your results are not good, then you have to keep seeing [your provider's] face.” (HIV[-], FGD, aged 25–35)

The perception that the clinical setting is synonymous with HIV is so strong that many women have described the “*the pressure for that place”* in both the IDI and FGD. Another participant explained the potential reaction of patients when knowing a community member has seen them in clinical settings as:

“You just hear someone say when I pass them ‘You see where that one went.’ I didn't tell them what I went [inside] for and they cause shame.” (HIV[-], IDI)

This concern is particularly strong that “*their neighbor might see”* (HIV[-], FGD, aged 46–55). Many of the statements from the HIV[-] participants indicate lack of privacy in their community, and that providers generally know their patients in social and community contexts outside the clinical setting. The participants described the potential to use outside providers to collect samples from patients or support patients who are self-collecting to encourage unscreened members of the community to participate. This was described further by one participant in the following statement:

“But from my experience, when you are talking to people, they say go [to the clinical setting] and I will open myself up to that provider. Then tomorrow the person can mock me … so clinical settings should take care to use people not familiar to the patients.” (HIV[-], FGD, aged 25–35)

In the HIV[-] group, there was some anxiety around the lack of familiarity with the clinical setting that was not discussed in the WLWH. Some of this included not being used to extended wait times, lack of familiarity with clinical procedures, lack of clarity on how they will receive their results and how to follow-up appointments. From the women who expressed these concerns, the majority responded that good hospitality and positive encouragement from staff would allow them to feel more comfortable in the clinical setting. As the level of anxiety for this group in accessing care in a clinical setting is high, many of them responded that similar procedures should be done together in a single visit:

“When you are sitting thinking going and taking your test, it's not easy to go. So when you have the opportunity to do one, if they can do all of it so that you can free your mind one time, it's very good.” (HIV[-], FGD, aged 25–35)

Regardless of HIV status, nearly all of the participants indicated that they or other women eligible for screening in their community had financial barriers that prevented them from seeking screening services and health care generally. One participant stated that women in their community would “*only to come to the hospital to do it if the money is available or if they will make it to be free.”* (HIV[-], FGD, aged 36–45). Another participant indicated the potential of free or lower cost screening services as a facilitator for an uptake in screening services in their community through the following statement: “*If someone said to me I can get the test done for free, I would go get it done myself so that I could experience it too.”* (WLWH, FGD, aged 36–45).

### The Influence of the Medical Provider on Collection Preferences

Several of the WLWH indicated their confidence in providers due to the status of their occupation. This was indicated through comments like “*… because it's their job”* (WLWH, FGD, aged 25–35), “*… the doctor is a specialist …”* (WLWH, IDI), and “*… because it's their work”* (WLWH, FGD, aged 25–35). In addition, the women who indicated that the provider's experience and knowledge is an indicator of why they prefer provider-collected sampling reported that: “*I tell the nurse [to collect my specimen] because I know they will do it better than me and then they will have it.”* (WLWH, FGD, aged 25–35) or “*… the nurse will know the right thing to do more than myself.”* (WLWH, FGD, aged 25–35).

Although the majority of the HIV[-] participants echoed these sentiments of confidence in their provider to collect their procedure, there was a small sub-group that had strong distrust and fear of their provider due to previous mistreatment in a clinical setting. One participant described an experience in which her provider mocked her body and repeatedly indicated that her “fatness” was getting in the way of procedures such as collecting blood and sitting on the exam chair. Another woman indicated a distrust of her sample being passed off to a provider whom she didn't know in the statement “*If it were another person that was sending it, you may not get to the root of the thing.”* (HIV[-], IDI) These two participants indicated that they would prefer self-collection if given the option, but also agreed they would allow a provider to collect their specimen despite their experiences with other providers.

### Beliefs About Consequences of Self-Collection

Many of the WLWH women cited that their level of comfort was a determining factor of their collection preference. The terms “shy” and “uncomfortable” were used by the participants to indicate women that were reluctant about sample collection in general. There were mixed results from this group on whether this discomfort encouraged them toward a preference of self- or provider-sampling.

“Some women will prefer to take it themselves because they are shy.” (WLWH, FGD, aged 46–59)

“So the reason that you use [provider] collection is if you don't feel comfortable putting [the collection swab] right at the cervix.” (WLWH, FGD, aged 25–35)

“Then [providers] will put the spatulum inside of women and the woman gets uncomfortable and can shake during [the procedure]” (WLWH, FGD, aged 25–35)

Among the HIV[-], this shyness was particularly surrounded in concern with privacy and exposing themselves to a provider. Generally, the women that described themselves or others as shy in this capacity indicated their preference for self-collection. One participant indicated that:

“Not everyone would like to open up herself for somebody to carry out a procedure on them. Some women are shy and they would not want to accept that somebody would see their nakedness.” (HIV[-], IDI).

The participants in both the HIV[-] and WLWH groups believed that self-collection would offer them more confidentiality than involving a provider in the collection process. One participant indicated that: “*I prefer collecting myself so that my secret should not be let out.”* (HIV[-], IDI). When asked how they interpret the preference of other women in their community in regards to privacy, they responded with comments such as “*… women will want to collect it themselves because they want to keep their result secret.”* (WLWH, IDIs) and “*… because they think it will be confidential.”* (WLWH, IDI).

## Discussion

The results of this study indicate that vaginal or cervical self-sampling is a well-accepted method that has the potential to increase participation in under screened populations in Limbé, Cameroon, which is reflected in literature on self-collection in similar low-resource settings (17–19). Although participants agreed on its acceptability, many of the participants, regardless of HIV status, did report that they prefer their provider collect specimens for HPV testing. The insight from both the IDI and FGD indicates that this is due to concern about their ability to self-collect and their own education on the procedure. Several other studies have demonstrated similar findings regarding our patients' concern for the reliability of self-collection (20, 21) and educational interventions decreased these concerns as well as increased patients' confidence in performing the self-sampling (22–24).

The participants in the WLWH group were less likely to have a preference for self-sampling, compared to the HIV[-] group. Our study found that WLWH indicated a strong reliance on providers for comfort and security through the procedure and within clinical settings. Ezechi et al. had similar findings that demonstrated the strong trust that exists between a WLWH and their provider and their influence in promoting positive health-seeking behaviors (25). Our recommendations include educational interventions tailored to providers who work with PLWH to educate and encourage patients on the importance of self-collection options. One study examined such an intervention and found that providers reported a higher importance of self-collection for HPV testing, an increase in knowledge to educate their patients on the procedure, and overall willingness to recommend self-collection (26).

Kumakech et al. asked several important questions regarding the impact of HIV stigma in selecting clinical sites associated with HIV treatment in the current policy push to integrate cervical cancer screening with current HIV care programs (27). The findings from our study indicate that a screening location that is known to be a site which offers HIV services may prevent HIV[-] women from seeking care. This concern may be linked to the study requirement to undergo an HIV test prior to enrollment, and the fear from HIV[-] women of being associated or diagnosed with HIV as a result. A scoping literature review by Stockton et al., supports our findings that HIV[-] women may fail to appear for screening services for fear of being associated with HIV (28). Therefore, we recommend further research to examine the role of HIV stigma in HIV[-] women in accessing cervical cancer screening in clinical sites to determine whether separate interventions need to be tailored based on HIV status.

At the time of publication, non-governmental actors that utilize external or international funding serve as the main source of access to cervical cancer prevention and treatment in Cameroon. Cervical cancer screenings in Cameroon should be focusing on development of government-led population-based program and policy initiatives that focus on reducing incidence of cervical cancer and out-of-pocket costs to patients. Based on our findings as well as lessons from similar settings, there is great importance in implementing structural reform in the country to ensure that women have access to appropriately tailored, cost-effective services for cervical cancer prevention, surveillance, and treatment.

## Conclusions

The findings presented in this paper demonstrate the acceptability of self-collection for specimens within our study community in Limbé, Cameroon. Given the resources available for the IDI and FDI, the type and number of participants selected for this study were limited and should be considered when examining the generalizability of these findings to women in other settings and contexts. However, the results indicate education and desensitization activities as facilitators and addressing the barriers of cost and HIV stigma in HIV[-] populations in implementation of self-collection strategies and campaigns for HPV testing across many LMIC settings, thus highlighting the urgent need to implement community-tailored interventions to reduce the burden of cervical cancer for women in Cameroon.

## Data Availability Statement

The raw data supporting the conclusions of this article will be made available by the authors, without undue reservation.

## Ethics Statement

The studies involving human participants were reviewed and approved by National Ethics Committee for Human Health Research (Cameroon) and Albert Einstein College of Medicine IRB (USA). The patients/participants provided their written informed consent to participate in this study.

## Author Contributions

PC, AA, RA, and KA conceptualized this project. AP conducted the analysis and drafted the manuscript with the guidance and assistance of AA. NF, JN, AD, and RN significantly contributed to the implementation of the study and facilitation of data collection in Limbé. All authors contributed to the article and approved the submitted version.

## Conflict of Interest

This study received Xpert HPV tests from Cepheid (Sunnyvale, CA, USA) at a reduced cost. PC, the PI of this study, has received HPV tests and assays for research from Roche, Becton Dickinson, Cepheid, and Arbor Vita Corporation at a reduced or no cost. The authors declare that the research was conducted in the absence of any commercial or financial relationships that could be construed as a potential conflict of interest.
